# Antenna Modification in a Fast-Growing Cyanobacterium Synechococcus elongatus UTEX 2973 Leads to Improved Efficiency and Carbon-Neutral Productivity

**DOI:** 10.1128/spectrum.00500-23

**Published:** 2023-06-15

**Authors:** Annesha Sengupta, Anindita Bandyopadhyay, Max G. Schubert, George M. Church, Himadri B. Pakrasi

**Affiliations:** a Department of Biology, Washington University, St. Louis, Missouri, USA; b Department of Genetics, Harvard Medical School, Boston, Massachusetts, USA; c Wyss Institute for Biologically Inspired Engineering, Harvard University, Boston, Massachusetts, USA; University of Michigan-Ann Arbor

**Keywords:** cyanobacteria, light harvesting, CRISPR-Cas3, antenna modification, sucrose production

## Abstract

Our planet is sustained by sunlight, the primary energy source made accessible to all life forms by photoautotrophs. Photoautotrophs are equipped with light-harvesting complexes (LHCs) that enable efficient capture of solar energy, particularly when light is limiting. However, under high light, LHCs can harvest photons in excess of the utilization capacity of cells, causing photodamage. This damaging effect is most evident when there is a disparity between the amount of light harvested and carbon available. Cells strive to circumvent this problem by dynamically adjusting the antenna structure in response to the changing light signals, a process known to be energetically expensive. Much emphasis has been laid on elucidating the relationship between antenna size and photosynthetic efficiency and identifying strategies to synthetically modify antennae for optimal light capture. Our study is an effort in this direction and investigates the possibility of modifying phycobilisomes, the LHCs present in cyanobacteria, the simplest of photoautotrophs. We systematically truncate the phycobilisomes of Synechococcus elongatus UTEX 2973, a widely studied, fast-growing model cyanobacterium and demonstrate that partial truncation of its antenna can lead to a growth advantage of up to 36% compared to the wild type and an increase in sucrose titer of up to 22%. In contrast, targeted deletion of the linker protein which connects the first phycocyanin rod to the core proved detrimental, indicating that the core alone is not enough, and it is essential to maintain a minimal rod-core structure for efficient light harvest and strain fitness.

**IMPORTANCE** Light energy is essential for the existence of life on this planet, and only photosynthetic organisms, equipped with light-harvesting antenna protein complexes, can capture this energy, making it readily accessible to all other life forms. However, these light-harvesting antennae are not designed to function optimally under extreme high light, a condition which can cause photodamage and significantly reduce photosynthetic productivity. In this study, we attempt to assess the optimal antenna structure for a fast-growing, high-light tolerant photosynthetic microbe with the goal of improving its productivity. Our findings provide concrete evidence that although the antenna complex is essential, antenna modification is a viable strategy to maximize strain performance under controlled growth conditions. This understanding can also be translated into identifying avenues to improve light harvesting efficiency in higher photoautotrophs.

## INTRODUCTION

Life on earth is sustained by solar energy and photosynthesis is the only known mechanism for life to harness the power of sunlight. Light harvested by photoautotrophs is used to convert atmospheric CO_2_ into sugar, and optimal cellular productivity necessitates a balance between the amount of light harvested by a cell and its ability to utilize the harvested light for carbon fixation (1). Under carbon-limited conditions, excess absorption of light can damage the photosynthetic apparatus and reduce productivity. All primary producers are equipped with light-harvesting complexes (LHCs) that enable efficient light capture (2). These antenna complexes originally evolved to improve photosynthetic efficiency under low light conditions that prevailed at the time of emergence of the first photosynthetic prokaryotes (2, 3). Therefore, though effective, and essential under light limiting conditions, these LHCs can become a liability when phototrophs are subjected to high light (3). Under extreme high light conditions, the excess light harvested by the LHCs, if not quenched, can damage the photosynthetic apparatus. Though this quenching process is energetically wasteful, it serves as a photoprotection mechanism to reduce photodamage (4). For example, under midday sunlight, organisms end up harvesting more light than can be utilized for CO_2_ fixation, despite some adjustment to the antenna structure. This leads to an imbalance in light absorption and carbon assimilation, eventually giving rise to harmful reactive oxygen species (ROS) and causing photodamage. Therefore, decades of research have focused on establishing the optimal antenna structure for maximal photosynthetic productivity under different growth conditions and several ideas have been proposed to circumvent this detrimental effect of the light harvesting antennae. Some of these include modifications to the structure or quantity of LHCs (5–7).

Cyanobacteria are the simplest photosynthetic organisms and have become the organisms of choice for understanding the complex process of photosynthesis due to ease of genetic manipulation and fast generation times (8). Cyanobacteria possess a special type of LHC called phycobilisomes (PBS). PBS is an array of protein-pigment complexes which in association with the photosystem (PS) reaction centers harness solar energy and drive cyanobacterial metabolism (9). Additionally, PBS strongly interacts with the ferredoxin-NADP(+) oxidoreductase-large protein (FNR_L_) which plays an important role in enabling cyclic electron transport for the generation of ATP and ensures efficient photosynthesis and CO_2_ assimilation in photoautotrophs. Previous studies have shown that the absence of FNR_L_ in mutant strains lead to inefficient cyclic electron flow (10, 11). The basic structure of the PBS antenna comprises the allophycocyanin core and the phycocyanin discs which are stacked into rod-like structures (12–14). The core and rods are assembled with the help of linker proteins that are not only involved in maintaining the structural integrity of the PBS molecules but also in transferring energy to the core (15). Though evolutionarily the most conserved component of PBS, the allophycocyanin core exhibits significant structural diversity and can be bi-, tri-, or penta-cylindrical (16, 17). While the highly studied tri-cylindrical core is found in most cyanobacteria and red algae (18–21), the less explored bi-cylindrical core has so far been identified only in Synechococcus elongatus, a clade which hosts some of the fastest growing strains identified to date (22–24). Unlike the strains with tri-cylindrical core PBS which possess a single copy of the phycocyanin genes (*cpcAB*) and two types of rod-core linker proteins (CpcG1, CpcG2), the strains possessing a bi-cylindrical core have two copies of the phycocyanin genes and one type of rod-core linker protein (CpcG) (25). Even the orange carotenoid protein (OCP) which interacts with CpcG1 and plays a role in photoprotection, is absent in *S. elongatus* strains (4, 26). In contrast to the stable PBS core, the rods are more dynamic in nature and are assembled and disassembled in response to light availability (17, 27). Altering the antenna size on demand is an energy intensive and inefficient process and therefore a burden on the cell (28, 29). Many studies in cyanobacterial strains, such as *Synechocystis* sp. PCC 6803, *Anabaena* sp. PCC 7120, and *Synechococcus* sp. PCC 7002 have attempted to understand the light harvesting mechanism and identify ways to optimize harvesting efficiency. Altering antenna size has been identified as a strategy to optimize light harvesting in photoautotrophs; the effectiveness of such a strategy is known to be strain-specific and differs considerably depending on the growth conditions used and the antenna component targeted (28, 30–35). For example, initial antenna modification studies in *Synechocystis* sp. PCC 6803, that used a low light growth condition, optimal for this strain, showed reduced growth and biomass production (32, 36, 37). Later studies with this strain that used higher light for growth demonstrated improved productivity with antenna modification (28, 35), although the relationship between such modifications and light and CO_2_ utilization remained inconclusive. One study concluded that high light and saturating CO_2_ conditions can lead to improved productivity in a strain with reduced antenna (28). Contradicting these findings, Lea-Smith et al., reported that high light and carbon limited conditions maximized the positive effect of antenna modification on the performance of strains with reduced antenna compared to wild type (WT) (35). Other antenna deletion studies in cyanobacterial strains did not investigate the impact of such modifications on strain productivity (15, 20, 21). More importantly, such studies have been confined to very few cyanobacterial strains and are completely lacking in high light tolerant strains that have been identified as potential platforms for bioproduction.

**FIG 1:**
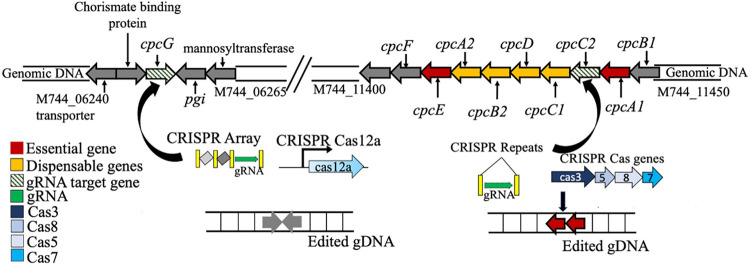
Schematics showing the location of the phycocyanin gene cluster in the genome of *S. elongatus* UTEX 2973. While most genes are clustered (*cpcB1*, *cpcA1*, *cpcC2*, *cpcC1*, *cpcD*, *cpcB2*, *cpcA2*, *cpcE*, *cpcF*), a single rod-core linker gene (*cpcG*) is localized distant from the cluster. The class I CRISPR/Cas system involves multiple Cas proteins (Cas3, Cas5, Cas8, Cas7, color scheme provided in the figure) and a specific gRNA (34-bp long, green) to achieve large deletion of dispensable regions. In this study, this class I CRISPR-Cas3-mediated deletion was employed for deleting most of the dispensable genes of the cluster by targeting a gRNA to *cpcC2* gene (green shaded). The resultant edited genome showed a deletion of 5 genes, approximately spanning a region of 4 kb and this strain is named Δrrl. On the other hand, the well-established class II CRISPR-Cas12a requires only one Cas protein, Cas12a, a gRNA (20 nucleotides in length, green) and a repair template to obtain targeted deletion. This system was used for targeted deletion of the isolated *cpcG* gene (green shaded), and the strain obtained is the ΔcpcG strain.

In this study, we investigated the effect of antenna modification on the photosynthetic efficiency and productivity of Synechococcus elongatus UTEX 2973, a fast-growing model cyanobacterium where rapid photosynthetic growth and high sucrose production titer have been reported (24, 38–40). We used a novel CRISPR-exonuclease system, CRISPR-Cas3 (41) in conjunction with the more widely used CRISPR-Cas12a (42, 43) to achieve various degrees of modification in the antenna structure. This strategy successfully deleted a region of 4 kb from the *S. elongatus* UTEX 2973 genome yielding the mutant Δrrl. This mutant lacks all rod-rod linker genes and one of the phycocyanin gene operons (Table 1, Fig. 1). Thus, the antenna structure in the Δrrl mutant comprises an intact core and one layer of phycocyanin disc attached to the core with the CpcG linker. We also attempted to delete the rod-core linker protein CpcG (no phycocyanin can attach to the core), to study the efficacy of the allophycocyanin core alone in driving energy transfer. To avoid the deletion of non-PBS genes in the vicinity of CpcG, an orphan gene residing in the genome of *S. elongatus* UTEX 2973 (Fig. 1), Cas12a-mediated gene-specific targeted deletion method was commissioned. Our results indicate that a truncated peripheral antenna is advantageous under ambient culture conditions of high light and atmospheric CO_2_ levels; however, complete disruption of the rods has a highly damaging effect on cellular growth. We further observed that the truncated peripheral antenna mutant (Δrrl) showed enhanced protein and sucrose production performance, demonstrating that antenna modification can be a viable strategy for optimizing light utilization and improving strain productivity.

**TABLE 1:** A section of the genetic annotations for *S. elongatus* UTEX 2973 showing the genes that are of interest for this study^*a*^

Locus tags of UTEX 2973	Annotation
M744_11400	Hypothetical protein
M744_11405	*cpcF*
M744_11410	cpcE*
M744_11415	*cpcA2*
M744_11420	*cpcB2*
M744_11425	*cpcD*
M744_11430	*cpcC1*
M744_11435	***cpcC2***
M744_11440	*cpcA1*
M744_11445	*cpcB1*
M744_11450	hypothetical

a The bold gene indicates the gene targeted to facilitate progressive deletion mediated by Cas3 nuclease. The asterisk (*) indicates an essential gene as per the essential gene list obtained for *S. elongatus* PCC 7942 (46). Underlined genes are the genes that were deleted using the CRISPR/Cas3 system with *cpcC2* as the target for gRNA.

## RESULTS

### Creating antenna mutants in Synechococcus elongatus UTEX 2973.

**FIG 2:**
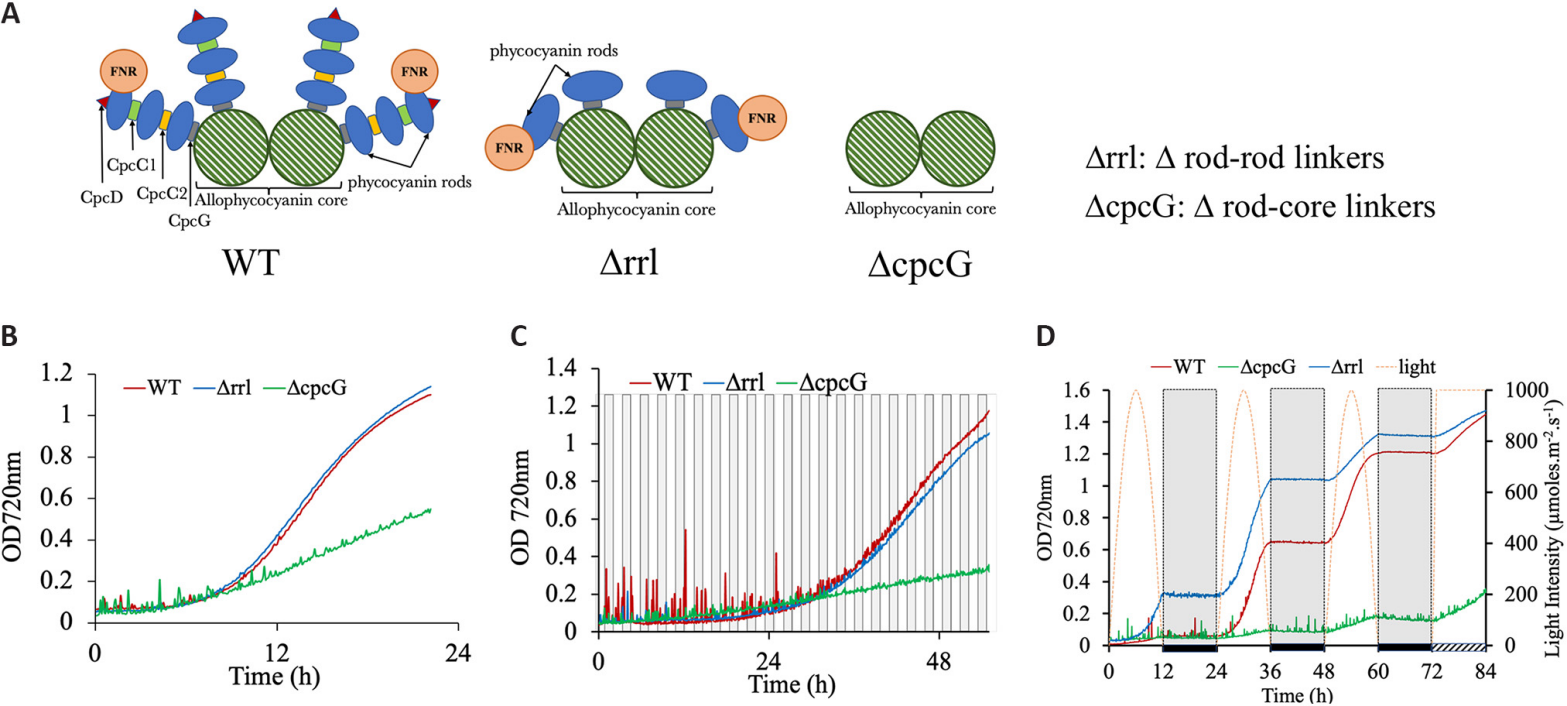
(A) Structure of phycobiliproteins in *Synechococcus* containing a bi-cylindrical core (green shaded) and phycocyanin discs (blue) stacked on to one another with the help of linker proteins (CpcC1, green; CpcC2, yellow; CpcD, red). The rod is attached to the core by linker protein CpcG (gray). The ferredoxin-NADP(+) oxidoreductase-large protein (FNR_L_ protein, brown) interacts with the phycocyanin rod to enable cyclic electron flow. CRISPR-mediated deletion generated antenna mutants: Δrrl and ΔcpcG. The cartoon diagrams are based on the available X-ray structures of phycobilisome. (B) Growth curve of WT, Δrrl, and ΔcpcG under HLHC (1,500 μmoles m^−2^ s^−1^, 1% CO_2_) in a multi-cultivator. (C) Growth profile of WT, Δrrl, and ΔcpcG under fluctuating high light of 1,500 μmoles m^−2^ s^−1^ (10m:10m ON-OFF) and 1% CO_2_ in a multi-cultivator. (D) Growth comparison of WT, Δrrl, and ΔcpcG under 12:12 h light-dark (LD) with sinusoidal illumination(1,500 μmoles m^−2^ s^−1^) and 1% CO_2_ condition in a multicultivator.

To determine the effect of antenna reduction on cyanobacterial fitness and performance, we generated two antenna mutants in *S. elongatus* UTEX 2973 using two different CRISPR Cas systems, Cas3 (41) and Cas12a (42). The inducible CRISPR/Cas3 system was adapted for use in cyanobacteria to achieve untargeted deletion of the maximal dispensable region of the phycocyanin gene cluster (Table 1, Fig. 1). Previous studies showed that the deletion of *cpcD*, the terminal linker, did not significantly reduce the rod length. However, deletion of *cpcC1* and *cpcC2* lead to a gradual reduction of antenna rod length, with the *cpcC2* deletion leaving only one phycocyanin disc attached to the core (44, 45). Therefore, in this study we targeted the *cpcC2* gene expecting deletion of nonessential genes in its vicinity. Approximately 20 transformants were induced for Cas expression and screening showed an exact deletion of 4 kb in all the transformants (see Fig. S1 and Table S1 in the supplemental material). The deleted region was further confirmed with whole-genome sequencing (WGS). WGS showed a clean deletion of the 3 rod-rod linkers (*cpcC1*, *cpcC2*, *cpcD*) and one of the copies of phycocyanin genes, *cpcB2A2* (Table 1). The analysis of WGS showed no additional mutations in the genome of the mutant. The deletion strain Δrrl (rod-rod linker mutant) lacks all the 3 rod-rod linkers and possesses only the rod-core linker (*cpcG*) which attaches to the core a single phycocyanin disc expressed from only one phycocyanin gene operon instead of two (Fig. 2A). This deleted region is flanked on one side by *cpcE*, a gene encoding a lyase understood to be essential (46), and on the other side by *cpcB1A1*, second copy of phycocyanin (Table 1). We attempted to delete these adjoining genes in both WT and Δrrl genetic background with CRISPR/Cas but were unsuccessful, suggesting their indispensability.

A second mutant was constructed using the established CRISPR/Cas12a method to delete the rod-core linker gene *cpcG* (Fig. 1). The ΔcpcG strain lacks the core-rod linker and therefore the rods cannot attach to the allophycocyanin core (Fig. 2A). PCR confirmation revealed a faint unsegregated band in the mutant (Fig. S2A), but RT-PCR analysis did not detect any transcripts (Fig. S2B), indicating that remnant copies of the gene are not expressed at detectable levels.

### Optimal antenna size is the key to improving light-harvesting capacity under high light conditions.

To determine the effect of antenna truncation in *S. elongatus* UTEX 2973, we first compared the growth profile of the WT, ΔcpcG, and Δrrl strains under optimal growth conditions of high light and high CO_2_ (HLHC). While the Δrrl strain showed a similar growth profile as the WT, the ΔcpcG strain exhibited a significant growth defect (Fig. 2B). Additionally, we tested the adaptability of these strains to fluctuating light intensity by subjecting them to 10 min of high light followed by 10 min of darkness. Though there was a prolonged lag in both the WT and Δrrl strains, they were able to adapt to the fluctuations. However, the ΔcpcG strain showed retarded growth under this condition (Fig. 2C and D). Even under 12 h light and 12 h dark condition, the ΔcpcG strain was unable to grow (Fig. 2D), indicating that the allophycocyanin core by itself was not sufficient and a phycocyanin disc linked by the *cpcG* gene to the core was essential for growth under these conditions. On the other hand, 12 h of sinusoidal illumination induced a longer lag in WT than the Δrrl strain (Fig. S3), but there was no difference in their final biomass accumulation (Fig. 2D). This longer lag in WT during the first cycle of growth can be attributed to lower cellular density that is more prone to photoinhibition, unlike the Δrrl strain, in which truncated antenna was advantageous.

**FIG 3:**
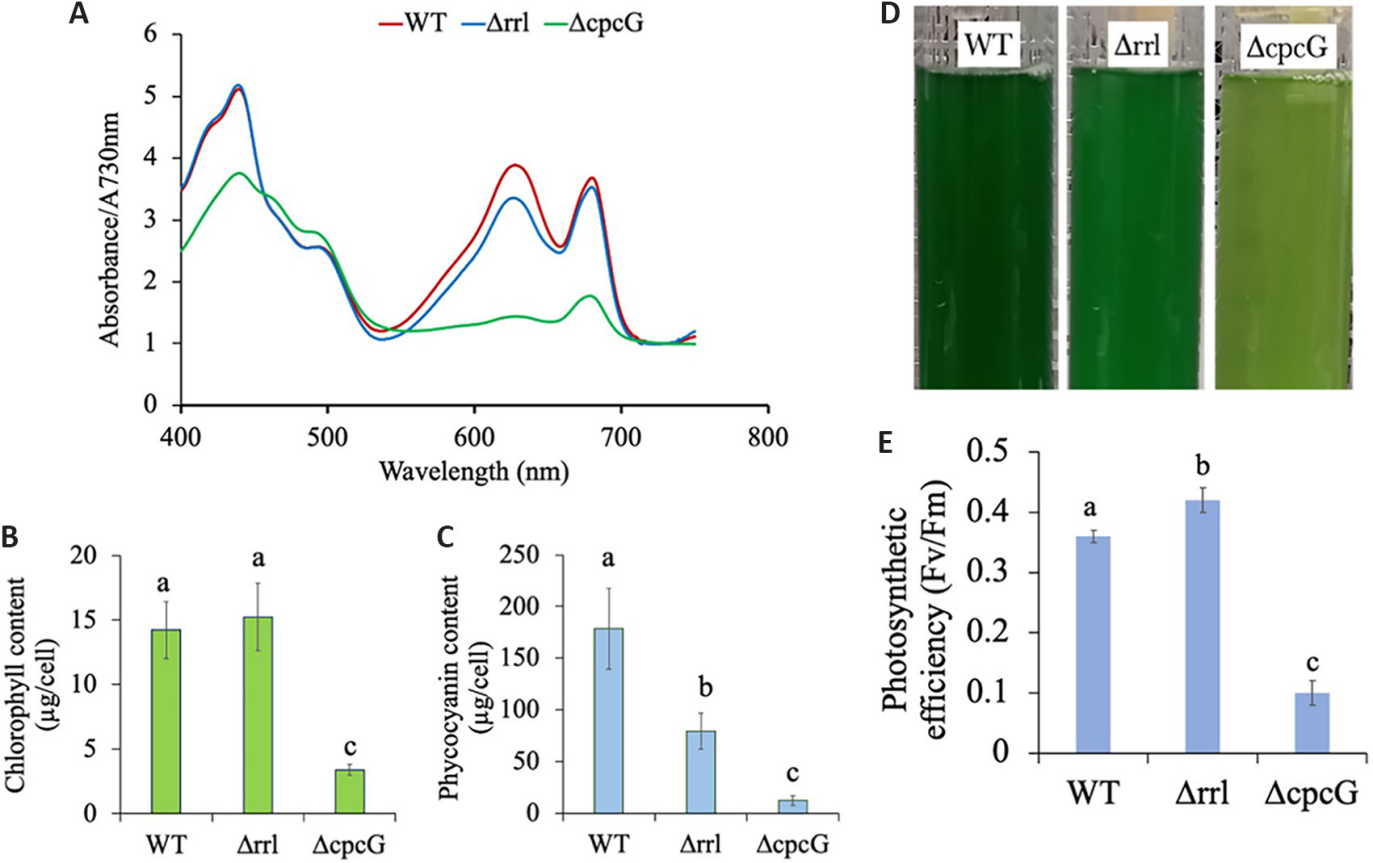
Comparison of photosynthetic parameters of strains when grown under HLHC in a multi-cultivator (A) Whole-cell absorption spectra. (B) Chlorophyll content determined from the whole-cell spectra. (C) Phycocyanin content estimated from the whole-cell spectra. (D) Phenotype differences of liquid cultures. (E) Photosynthetic efficiency of the strains. Error bars correspond to standard deviations from at least three biological replicates. The same letters denote statistically insignificant different values of μ, while different letters (a to c) are given for statistically significant μ for each category (*P* < 0.05).

The whole-cell absorption spectra for the strains (WT, Δrrl, and ΔcpcG) were obtained to estimate the chlorophyll and phycocyanin levels under HLHC (Fig. 3A). While the WT and Δrrl strains have similar levels of chlorophyll, the ΔcpcG strain shows significantly lower chlorophyll levels (Fig. 3B). On the other hand, estimation of whole-cell phycocyanin content showed a trend that was expected, with WT showing the highest level of phycocyanin, Δrrl exhibited a reduction in phycocyanin levels by 55% and a significant reduction of ~93% was observed in ΔcpcG strains (Fig. 3C). This sequential reduction in phycocyanin content in the mutants was also evident from the differential bleaching phenotype of the culture. The ΔcpcG strain showed the most bleached phenotype compared to the WT or Δrrl (Fig. 3D). 77 K Fluorescent spectra (580 nm excitation) revealed a prominent peak shift to 658 nm in both ΔcpcG and Δrrl strains unlike the 650 nm peak in WT, signifying the predominance of allophycocyanin over phycocyanin in the mutants (Fig. S4). We also estimated the photosynthetic efficiency of the strains (F_v_/F_m_) and found that the ΔcpcG strain exhibited significantly lower efficiency than the other strains (Fig. 3E). As seen in Fig. S5, the 695/685 ratio, a signature of functional PSII, is skewed in the ΔcpcG mutant suggesting that a significant fraction of the PSII pool might be inactive. Further, the peaks representing energy transfer to the photosystems (arrows in Fig. S4A) were absent in the ΔcpcG strain, an observation in agreement with the reduced photosynthetic efficiency of this strain. These studies demonstrated that while the inability of the rods to assemble onto the core can be detrimental to cell growth, a single layer of phycocyanin disc attached to the core can be sufficient for growth under optimal conditions.

### Δrrl outperforms the WT when grown under high light and air.

**FIG 4:**
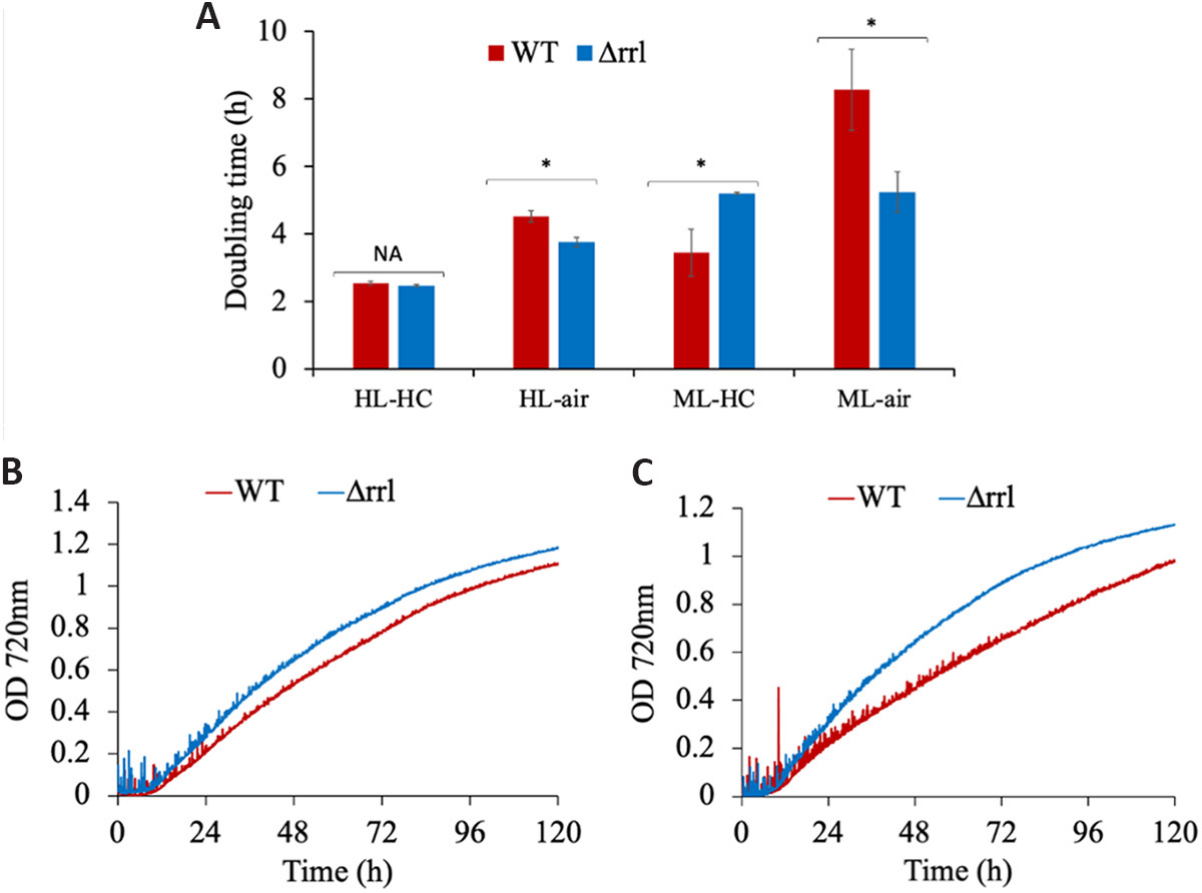
Growth comparison of WT and Δrrl under specified conditions (A) Doubling time comparison under different growth conditions of light and CO_2_: HLHC (1,500 μmoles m^−2^ s^−1^, 1% CO_2_), HL-air (1,500 μmoles m^−2^ s^−1^, air), MLHC (800 μmoles m^−2^ s^−1^, 1% CO_2_), ML-air (800 μmoles m^−2^ s^−1^, air). (B) Growth profiles of the strains under HL-air. (C) Growth profiles of the strains under ML-air. Error bars correspond to standard deviations from at least three biological replicates. While NA indicates statistically insignificant μ, an asterisk (*) denotes values of μ are statistically different for each category (*P* < 0.05).

The surprising similarity of Δrrl’s growth characteristics to WT prompted us to further probe its performance under diverse growth conditions. When cultivated under low light conditions, the mutant exhibited retarded growth, suggesting that the reduced antenna is insufficient in harvesting optimal quantity of light (Fig. S6). We also compared the growth of the Δrrl strain to that of the WT under two different light conditions supplemented with either high or ambient CO_2_ levels (Fig. 4A). Intriguingly, we found that the growth of the mutant was enhanced by up to 36% under ambient air conditions compared to the WT (Fig. 4). Analysis of the growth profiles indicated that while the WT exhibited significant growth retardation under ambient air, the mutant did not (Fig. 4).

**FIG 5:**
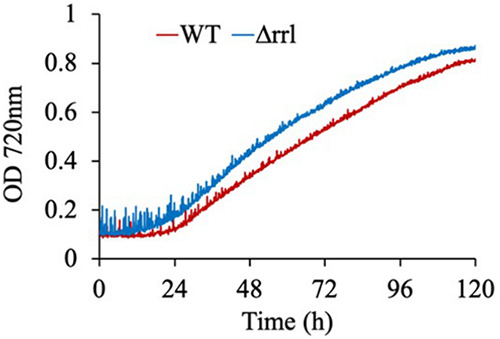
Growth comparison between WT and Δrrl under 10 min ON and 10 min OFF of 1,500 μmoles m^−2^ s^−1^ light with bubbled air.

We repeated the strain adaptability study under fluctuating light and dark conditions and ambient air. Once again, the mutant outperformed the WT (Fig. 5). This demonstrates that antenna reduction confers significant growth advantage to cells growing under ambient air when light is not a limiting factor and also enhances the strain’s ability to adapt to fluctuating light conditions.

### Antenna modification leads to improved productivity.

**FIG 6:**
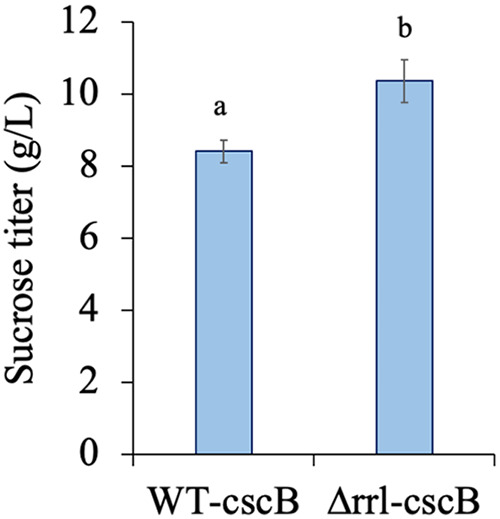
Productivity of WT and Δrrl strains: sucrose accumulation under optimal production condition. Error bars correspond to standard deviations from at least three biological replicates. The letters (a and b) denote statistically different values of μ for each category (*P* < 0.05).

The improved fitness of the Δrrl strain motivated us to test its efficacy as a host for protein production. We initially measured the total protein from the WT and Δrrl strains (grown to equal cell density). Since total protein estimation showed 14% ± 4% higher protein content in Δrrl strain than the WT, we proceeded to test the levels of a heterologous reporter system, enhanced yellow fluorescent protein (eYFP), a stable reporter protein that is not regulated by cellular metabolism and is easy to detect (47). Interestingly, eYFP levels were strikingly higher (~5-fold) in the mutant (Δrrl strain expressing eYFP) compared to the WT strain. We further tested the performance of this strain by expressing the sucrose transporter (*cscB*) and compared its sucrose producing ability to the previously published sucrose producing *S. elongatus* UTEX 2973 strain (40). Echoing previous results, sucrose overproducing strains of both WT and ΔrrL backgrounds grew similarly in HL-HC conditions, and in ambient air conditions the Δrrl strain grew more quickly (Fig. S7). Estimation of the sucrose accumulation in the media indicated a 22% increment in levels for the Δrrl strains compared to the engineered strain (40) (Fig. 6). The sucrose titer observed in the antenna mutant is the highest observed in cyanobacteria to our knowledge.

## DISCUSSION

Photosynthetic organisms are equipped with LHCs to harness the power of sunlight. Though sunlight is essential for sustenance, too much light energy can be detrimental for photoautotrophic growth and productivity due to the formation of harmful ROS. Elevated CO_2_ levels can alleviate this effect to some extent by channelizing the excess light energy into the carbon fixation pathway, leading to higher biomass accumulation. However, under high light and ambient atmospheric CO_2_ levels the light energy absorbed exceeds the rate of carbon utilization, causing photodamage. This phenomenon is a major problem that affects global productivity and consequently has sought the attention of the scientific community for decades (5). There has been a long-standing hypothesis that a smaller antenna size might provide added advantage on cellular growth and productivity under artificial, prolonged high light conditions where larger antennae can cause photobleaching (27, 28, 48, 49). In agreement with this hypothesis, the fast-growing strain *Synechococcus* PCC 11901 identified from a tropical waterbody that is exposed to high light intensities has reduced antenna complex (49).

In this study, we investigated the feasibility of improving photosynthetic productivity by altering the antenna structure of *S. elongatus* UTEX 2973, a strain known for its fast growth characteristics and high light tolerance (24). These characteristic features of *S. elongatus* UTEX 2973 make the strain best suited for studying the antenna modification under conditions which cause photodamage. *S. elongatus* UTEX 2973 exhibits high photosynthetic efficiency and carbon fixation rates and has immense potential to be developed as a green production platform (38, 47, 50–52). Many studies have already taken up this challenge and demonstrated higher productivity of this strain compared to other cyanobacterial hosts (40, 52). However, to make it commercially competitive, more avenues of improving its productivity need to be explored and antenna adjustment is an attempt in that direction. Recent availability of the detailed X-ray crystal structures of tri-cylindrical PBS (PDB IDs: 7SC8, 7SC7, 7SC9, 7SCB, 7SCC, 7SCA) from *Synechocystis* sp. PCC 6803 and bi-cylindrical core (PDB ID: 4F0U) and phycocyanin (PDB ID: 4H0M) from *S. elongatus* PCC 7942 have provided us a basis to design levels of deletions for improved understanding of cyanobacterial light harvesting (4, 12). Fig. 2A shows a diagram of the PBS structure in the WT and the antenna deletion mutants, assembled based on information obtained from the PDB database. It depicts how a systematic deletion of the genes impacts the structure of the antenna in the mutants (Δrrl and ΔcpcG) compared to the WT. In the Δrrl strain, all the rod-rod linkers and one of the two copies of the phycocyanin genes have been deleted, thereby leaving a truncated peripheral antenna with an intact CpcG linker protein and a single layer of phycocyanin which can interact with FNR_L_ (Fig. 2A). ΔcpcG is devoid of the rod-core linker and therefore rods formed cannot assemble over the allophycocyanin core leaving the core as the only light capturing unit in the antenna of this strain with no provision for FNR_L_ to interact with PBS (Fig. 2A). These two mutants help us systematically decipher the correlation between antenna structure and productivity.

A novel CRISPR-based genome editing tool was adapted and used for the first time in cyanobacteria to assess the maximum extent of deletion possible in the PBS cluster (Fig. 1). This led to a strain with a deletion of 4 kb (Δrrl), a region which spanned between *cpcE* and *cpcB1A1* genes. Several attempts to extend this region beyond 4 kb were unsuccessful. The *cpcE* gene has been identified as an essential gene in *S. elongatus* PCC 7942 (46), and this essentiality likely restricted Cas3-helicase-nuclease from progressing further in that direction. The inability to delete *cpcB1A1* indicates the importance of retaining at least a copy of the phycocyanin gene in *S. elongatus* 2973.

The growth and phenotype analysis of the mutants demonstrated that just the core is not enough for light harvest and a basal antenna structure with at least a single phycocyanin disc attached to the core mediated by the CpcG linker is essential even under high light and high CO_2_ (HLHC) conditions (Fig. 2B). This basal antenna structure is even more critical under fluctuating light, a condition cells encounter outdoors, as evident from the highly impaired growth of the ΔcpcG strain (Fig. 2C and D). Though the phycocyanin genes were not deleted from the ΔcpcG strain, it exhibited a severely bleached phenotype and reduced phycocyanin content (Fig. 3D), indicating that the pigment, though synthesized, is not functional and probably degraded (Fig. 3A). Therefore, FNR_L_, which interacts with PBS to perform cyclic electron transport, is also nonfunctional in the ΔcpcG strain. The ΔcpcG strain also exhibited lower photosynthetic efficiency (Fig. 3E), indicating that suboptimal light harvesting, compromised photoprotection, and inefficient FNR_L_ activity in the absence of a rod-core linker and phycocyanin rods can affect photosynthesis and growth. While complete disruption of the rod-core structure is detrimental, the presence of only one disc of phycocyanin associated with the core is enough for the cells of high light tolerant strains like that of *S. elongatus* UTEX 2973, to perform at their optimal level under HLHC, as seen in the Δrrl strain (Fig. 2B). Unlike the ΔcpcG strain, the presence of single layer of phycocyanin attached to the core with the help of CpcG linker in Δrrl strain allowed the interaction of the FNR_L_ protein with PBS (11) and hence the mutant exhibited a WT-like photosynthetic signature (Fig. S4B) though the phycocyanin levels were reduced as observed in 77 K fluorescence analysis. It shows that reduction in antenna size of a bi-cylindrical core PBS is advantageous to an extent that it does not hamper photoprotection or energy transfer to the PS. In a previous study, deletion of the entire cpc operon (Δ*cpcBcpcAcpcC1cpcC2cpcD*) in *Synechocystis* sp. PCC 6803 gave rise to a strain that showed a PBS architecture CK-like (10) (where the strain is devoid of phycocyanin, but the CpcG is present on the core) instead of Δrrl-like (Fig. 2A) or CB-like (10). The *Synechocystis* sp. PCC 6803 Δcpc mutant showed improved biomass accumulation compared to WT under high light conditions, though the growth rates were comparable with low levels of chlorophyll (28). Unlike the bicylindrical core of *S. elongatus* UTEX 2973, *Synechocystis* sp. PCC 6803 has a tri-cylindrical allophycocyanin core with attached rods and the differences in phenotype observed in the respective mutant strains might be attributed to the difference in the core (bi-cylindrical versus tri-cylindrical) structure (16, 17). Fig. S9 provides a consolidated picture of the effect of antenna modification on strains with (a) bi (current study) or (b) tri-cylindrical (from existing literature) core architecture. This is the first report of antenna modification in a strain with bi-cylindrical core. In both types of PBS, reduction in phycocyanin rod length lowers photoinhibition and optimizes net light harvesting, making the structure advantageous under extreme high light (35) (Fig. 2B) but not under low-light intensities (32) (Fig. S6). Complete deletion of phycocyanin in strains with tri-cylindrical core resulted in improved biomass accumulation. This suggests that the presence of rod-core linker which binds OCP and mediates photoprotection is enough to maintain strain fitness, though the efficiency is lower than structure containing at least one phycocyanin disc (28, 35). However, a similar deletion has not been reported in strains with a bi-cylindrical core, and our attempts to generate such a strain were also not successful. Further deletions that leave the core alone prove detrimental for growth phenotype (20, 25, 53). This indicates that antenna reduction can improve photosynthetic efficiency and strain fitness under specific growth conditions if the photoprotection mechanism is not compromised.

**FIG 7:**
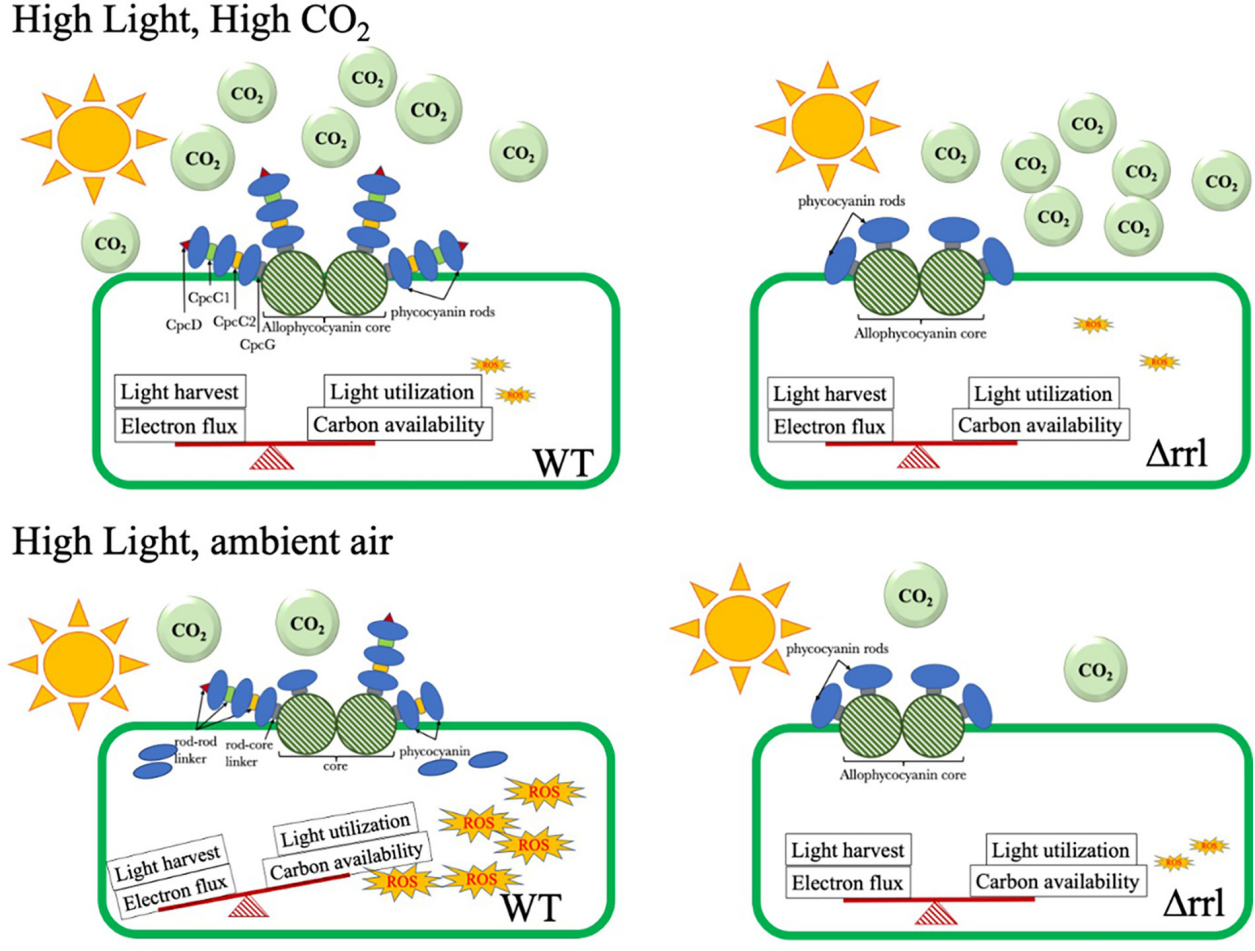
Model showing the effect of antenna modification on cell fitness when sufficient light is available with either higher CO_2_ or ambient air.

Interestingly, the Δrrl mutant considerably outperformed the WT strain under high light and ambient air as well as under intermittent high light and air conditions that mimic the outdoor environment significantly (Fig. 4 and 5). The mutant showed up to 36% improvement in growth compared to the WT under high light and ambient air. A similar observation was reported earlier, where different antenna mutants outperformed under low carbon compared to high CO_2_ levels (30, 35). This improved fitness of the mutant compared to the WT under low carbon and high light can be explained with the help of the model in Fig. 7. The model proposes that under high CO_2_ and high light condition, light absorption synchronizes with carbon assimilation. Since *S. elongatus* UTEX 2973 exhibits a high CO_2_ sequestration rate, the excess light energy absorbed under extreme high light conditions is totally utilized for CO_2_ fixation, preventing photodamage. However, under high light and ambient carbon levels there is a dramatic disparity between light absorption and utilization in the WT. This leads to the production of ROS and eventually causes photodamage and growth retardation. On the other hand, the minimized antenna in Δrrl strain is advantageous under high light intensity and both low and high carbon conditions because it restricts the absorption of excess light energy when the cells are unable to process it. In addition, the excess energy spent in the not so efficient process of dismantling and assembling the antenna in the WT is avoided in the mutant. These advantages in the mutant are also reflected in its ability to synthesize larger number of total proteins (14% ± 4), reporter protein (eYFP) and products (sucrose) compared to the WT (Fig. 6).

In conclusion, our work demonstrates that modifying the LHCs strategically can lead to significant advantages in growth and productivity. Introducing a minor alteration in the initial steps of photosynthesis can translate into significant improvement as observed in the 22% increase in sugar production in the Δrrl strain. These findings can be implemented to engineer industrially relevant phototrophs for superior performance under ambient growth conditions that induces photodamage in cells.

## MATERIALS AND METHODS

### Chemicals and reagents.

All enzymes were purchased from New England Biolabs (NEB, Ipswich, MA, USA) and ThermoFisher Scientific (Waltham, MA, USA). The isolation and purification kits were obtained from Sigma-Aldrich (St. Louis, MO, USA). All chemicals, reagents and antibiotics used were of analytical/HPLC grade and were procured from Sigma-Aldrich (St. Louis, MO, USA). The primers were ordered from Integrated DNA Technologies (IDT, Coralville, IA, USA). The plasmids were sequenced by Genewiz (South Plainfield, NJ, USA).

### Cultivation condition of the WT and mutants.

The WT *S. elongatus* UTEX 2973 strain and mutant strains 2973ΔcpcG and 2973Δrrl were cultivated and maintained in Caron chamber under 300 μmoles m^−2^ s^−1^ light and 1% CO_2_ at 38°C with orbital rotation of 250 rpm. E. coli containing specific plasmids was cultivated overnight at 37°C in LB supplemented with appropriate antibiotic. Freezer stocks were maintained at −80°C in 7% dimethyl sulfoxide (DMSO) for cyanobacterial strains and 25% glycerol for E. coli strains.

### Strain development strategies.

Two genome editing techniques were employed for obtaining the phycocyanin mutants in *S. elongatus* UTEX 2973: Cas3-mediated progressive deletion and Cas12a-meditated directed deletion. The protocols in brief are provided:

**(i) Cas12a-mediated strategy.** The ΔcpcG strain was obtained by Cas12a CRISPR method, where a specific gRNA was targeted to the *cpcG* gene (M744_06250) of *S. elongatus* UTEX 2973. The gRNA and the repair template (750 bp upstream and downstream sequence of M744_06250 were fused to form the repair template) were cloned into the replicative editing vector pSL2680 (42) by Gibson assembly to generate the recombinant plasmid pSL3342. The plasmid was maintained in E. coli XL1-Blue strain. The plasmid was sequenced from Genewiz. The pSL3342 plasmid was transformed into *S. elongatus* UTEX 2973 by triparental mating (42) using the conjugal plasmid pRL443 (54) to generate the ΔcpcG strain. Posttransformation, the strain was patched on kanamycin plate and checked for segregation by PCR using confirmation primers specific for the locus region outside the repair template. All primers used in this study are listed in Table S1. ΔcpcG strain was cultivated in BG11 supplemented with kanamycin.

**(ii) Cas3-mediated strategy.** The Δrrl strain was constructed using a CRISPR technique novel to cyanobacterial system. The CRISPR-Cas3 is a class I CRISPR system that employs multiple Cas proteins for its functioning (41). The plasmid containing the *cas* genes was procured from Addgene. These *cas* genes (*cas3*, *cas5*, *cas7*, and *cas8*) along with the CRISPR repeats were assembled on to the RSF1010 plasmid background by Gibson assembly to generate a recombinant plasmid pSL3577 (pRhRB-Cas3578). The expression of *cas* genes and the gRNA were controlled using the rhamnose inducible promoter and theophylline inducible riboswitch. A gRNA targeting the *cpcC2* gene (M744_11435) was cloned on to the BsaI restriction site and unlike Cas12a, this system does not require any predetermined repair template. The recombinant plasmid (pSL3578) was conjugated into the *S. elongatus* UTEX 2973 by triparental mating using the protocol described previously (42) and selected against kanamycin resistance. A transformant colony was grown in 20 mL BG11+Kan media and induced with 2g/L rhamnose and 1 mM theophylline for 24 h to obtain progressive deletion. The induced culture was diluted and plated on a kanamycin plate to obtain single colonies. These colonies were tested for deletion using tiling PCR (41). Primers are listed in Table S1. All the colonies tested showed a deletion of 4 kb. The deleted strain (Δrrl) was cured of the editing plasmid and grown for genomic DNA isolation (55) and whole-genome sequencing (WGS) to confirm the deletion and identify additional mutations. WGS was performed as per Baym et al. (56). Briefly, a scaled-down adaptation of the Nextera (Illumina) protocol was performed, in which DNA is “tagmented” by tn5 transposase, and sequencing indexes are added by PCR. PCR was performed with Q5 polymerase (New England Biolabs) and indexing primers previously described (56) and amplified for a total of 15 cycles. Resulting amplicons were purified and sequenced on the MiSeq instrument (Illumina). Analysis of resulting sequences was performed using Breseq (57).

### RNA isolation and RT-PCR of the mutant and WT.

The WT and ΔcpcG strains were cultivated in 1% CO_2_ and high light intensity to exponential phase of an optical density at 730 nm (OD_730_) of 0.6. Then, 50 mL of the culture was harvested and the total RNA was isolated using the isolation kit (Sigma, USA), which was further converted to cDNA by the iScript advanced cDNA synthesis kit from Bio-Rad following the manufacturer’s instructions. Consequently, the comparative transcript level of *cpcG* was visualized on a gel via qualitative RT-PCR from the synthesized cDNA using primers RT-cpcG-fw/RT-cpcG-rv (Table S1).

### Growth experiments and photosynthetic parameter measurements.

The WT and the mutant strains were grown at 38°C in 100 mL glass cultivation tubes of Multi-Cultivator Photobioreactor (Photon Systems Instrument, Multi-Cultivator MC 1000, Czech Republic) containing 50 mL BG11 media under different light intensities 100 (low light [LL]), 800 (medium light [ML]), and 1,500 (high light [HL]) μmoles m^−2^ s^−1^. The aeration provided for growth was by bubbling either 1% CO_2_ mixed air or atmospheric air through the culture. Cells were grown in a shake flask for a day under 300 μmoles m^−2^ s^−1^ light and 1% CO_2_ at 38°C with an orbital rotation of 250 rpm and these cells were used as the starter inoculum for the experiment. These cells were inoculated at an OD_720_ of 0.025 to 0.1 and were cultivated to an OD_720_ of 1 or 1.5. The growth rate and doubling time was calculated in the exponential phase of growth. All experiments were performed in biological replicates (at least *n* = 3), and the data shown is an average of the replicates and the error bars correspond to standard error of mean (SEM) of the biological replicates (at least *n* = 3).

The absorption spectra of the WT and mutant strains under different growth conditions were obtained at room temperature using Shimadzu UV-1800 spectrophotometer. The whole-cell phycocyanin and chlorophyll contents were calculated from the absorption spectra using the formulae obtained from Arnon et al. (58). The fluorescence emission spectra of phycobilisome from the whole cell of each strain were measured at 77 K using a FluoroMax-2 fluorometer (Jobin Yvon). Phycocyanin was excited at 590 nm, and fluorescence emission was recorded between 620 nm and 750 nm and normalized at 730 nm. The measurements were made on a SPEX Fluoromax 2 spectrofluorimeter and analyzed with Data Max for Windows. The cultivated cyanobacterial cells were normalized based on chlorophyll content to determine the quantum efficiency (59) of strains grown under different growth conditions using the FL-200 dual modulation PAM fluorometer with blue light activation as per previous protocol (26). The efficiency is calculated as F_v_/F_m_ = (F_m_ – F_0_)/F_m_, where F_0_ is the minimum fluorescence, F_v_ is the variable fluorescence, and F_m_ is the maximum fluorescence.

To determine whether the mutants and WT were able to adapt to fluctuating light conditions, both the strains were cultivated in a multi-cultivator (MC) at 38°C bubbled with either 1% CO_2_ blended air or atmospheric air for a period of 70 h or 120 h, respectively. The light intensity used for these studies were set at 1,500 μmoles m^−2^ s^−1^, which was subjected to two cycles: (i) 10 min ON and 10 min OFF cycle, (ii) 12 h of light (light intensity subjected to sinusoidal function) and 12 h of dark for the duration of growth period. These experiments were also performed in biological replicates (*n* = 3).

### Estimation of total protein content and eYFP levels.

The total protein was extracted from the WT and Δrrl strains when grown to a midexponential phase under optimal growth conditions and estimated using the bicinchoninic acid (BCA) protein assay reagent (Thermo Scientific). The experiment was performed in triplicates.

To measure the productivity using a reporter system (enhanced yellow fluorescent protein [eYFP]), a replicative plasmid containing the *eyfp* gene under the control of a native promoter (47, 60) was transformed in WT and Δrrl strains. The transformants were cultivated under optimal growth conditions and eYPF fluorescence was estimated in the midexponential phase using the Bio-Tek μQuant plate reader, $\lambda_{\text{ex}}$ = 514 nm, and $\lambda_{\text{em}}$ at 527 nm (47). Experiments were performed in biological (*n* = 3) and technical (*n* = 3) replicates.

### Introducing the *cscB* gene for sucrose overproduction and sucrose estimation.

The plasmid containing the *cscB* gene encoding sucrose permease from E. coli (ATCC 700927) was previously modified to be integrated at the neutral site 3 (NS3) of *S. elongatus* UTEX 2973 (40). In this study, to determine the sucrose productivity, we transformed the plasmid and ensured integration of the *cscB* gene into the mutant Δrrl strain at NS3 and then compared it to the highest sucrose-producing strain of *S. elongatus* UTEX 2973, previously reported (40). To determine sucrose levels previous protocol was followed, briefly, the strains were cultivated in BG11 media supplemented with 150 mM NaCl in a Caron chamber at 38°C, 300 μmoles m^−2^ s^−1^ light and 0.5% CO_2_ for 3 days and sucrose in the supernatant was measured using the sucrose/d-glucose assay kit (Megazyme) (40). The standard curve for sucrose and glucose were performed (Fig. S8). The experiment was performed in biological (*n* = 3) and technical (*n* = 3) replicates.
