# Erratum for Sengupta et al., “Antenna Modification in a Fast-Growing Cyanobacterium *Synechococcus elongatus* UTEX 2973 Leads to Improved Efficiency and Carbon-Neutral Productivity”

**DOI:** 10.1128/spectrum.00887-26

**Published:** 2026-07-10

**Authors:** Annesha Sengupta, Anindita Bandyopadhyay, Max G. Schubert, George M. Church, Himadri B. Pakrasi

## ERRATUM

Volume 11, no. 4, e00500-23, 2023, https://doi.org/10.1128/spectrum.00500-23. Acknowledgments, last sentence: “…see v.ht/PHNc” should read “…see arep.med.harvard.edu/t.”

