# Supplementary material for: Antenna Modification in a Fast-Growing Cyanobacterium Synechococcus elongatus UTEX 2973 Leads to Improved Efficiency and Carbon-Neutral Productivity
Source: Microbiol Spectr. 2023 Jun 15;11(4):e00500-23. doi: 10.1128/spectrum.00500-23 (PMC10433846; doi:10.1128/spectrum.00500-23)
Supplement: Supplemental file 1 — Supplemental material. Download spectrum.00500-23-s0001.pdf, PDF file, 0.7 MB [file spectrum.00500-23-s0001.pdf]

Supplementary Information:

Antenna modification in a fast-growing cyanobacterium *Synechococcus elongatus* UTEX 2973 leads to improved efficiency and carbon-neutral productivity

Annesha Sengupta[a], Anindita Bandyopadhyay[a], Max G. Schubert[b,c], George M. Church[b,c], Himadri B. Pakrasi[a,#]

[a]Department of Biology, Washington University, St. Louis, MO, USA;

[b]Department of Genetics, Harvard Medical School, Boston, MA, USA;

[c]Wyss Institute for Biologically Inspired Engineering, Harvard University, Boston, MA, USA.

[#]Corresponding author: Himadri B. Pakrasi, pakrasi@wustl.edu.

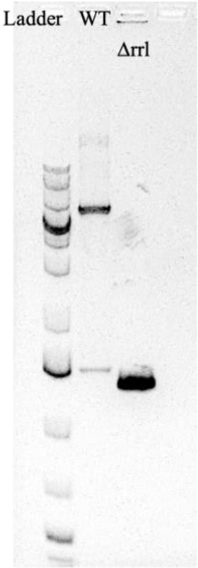

Fig. S1: Gel picture showing the deletion confirmation of 4 kb in Δrrl strain as compared to WT.

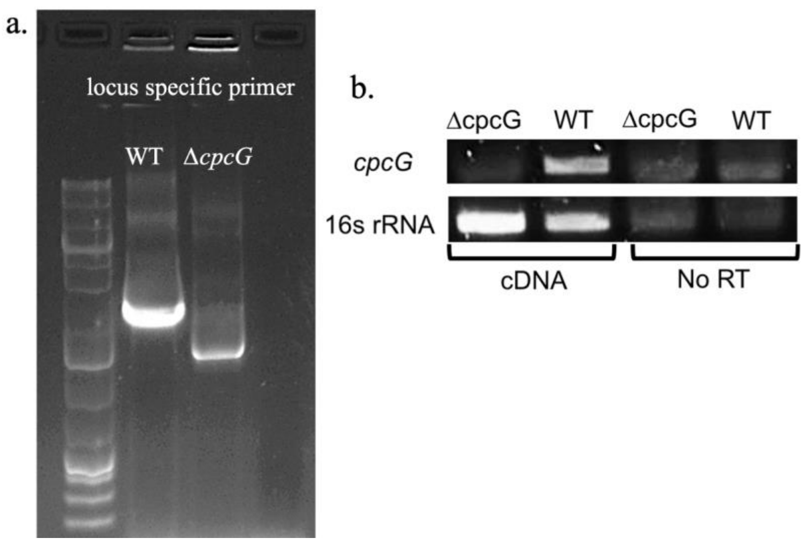

Fig. S2: a. The PCR confirmation of the ΔcpcG strain. The locus specific primers for the ΔcpcG shows one band showing the deletion of *cpcG* gene and a faint band corresponding to the WT band indicating an unsegregated copy of *cpcG* gene. b. The RT-PCR gel picture showing no transcript for ΔcpcG mutant as compared to the WT.

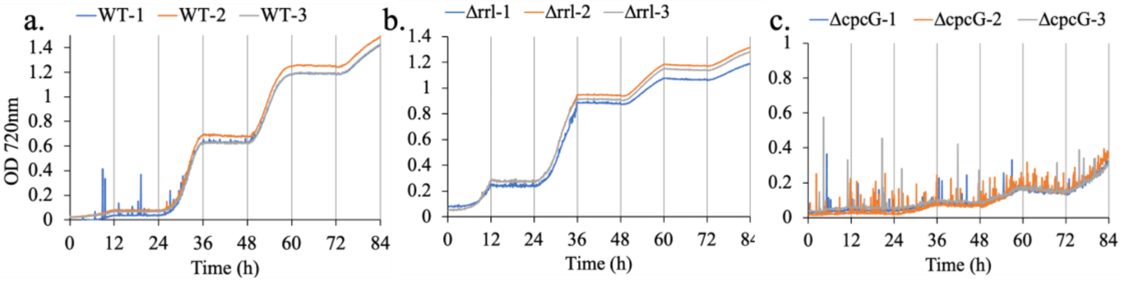

Fig. S3: Growth curve (showing all three replicates) of WT, Δrrl and ΔcpcG strains when grown under 12h of light and 12h of dark (12:12h LD) cycle for a period of 84 h. The 12h of illumination was programmed to a sinusoidal function.

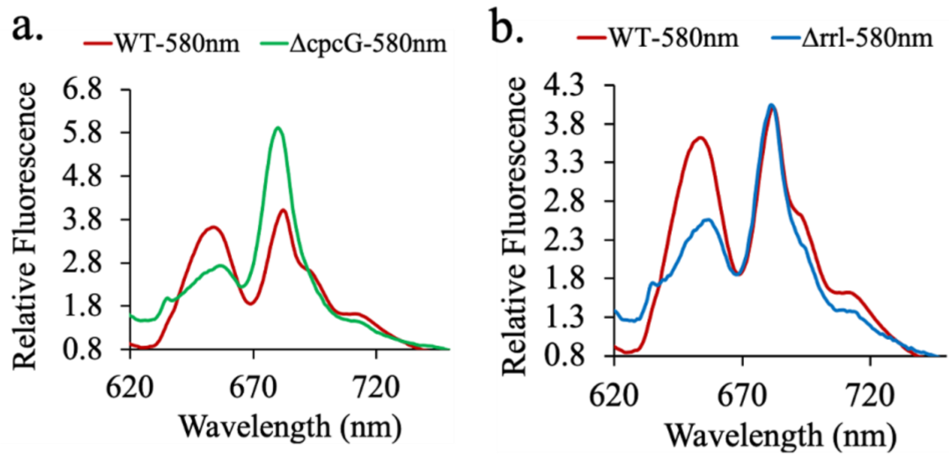

Fig. S4: 77K Fluorescence emission spectra of the WT, Δrrl and ΔcpcG strains when excited for phycocyanin at 580nm.

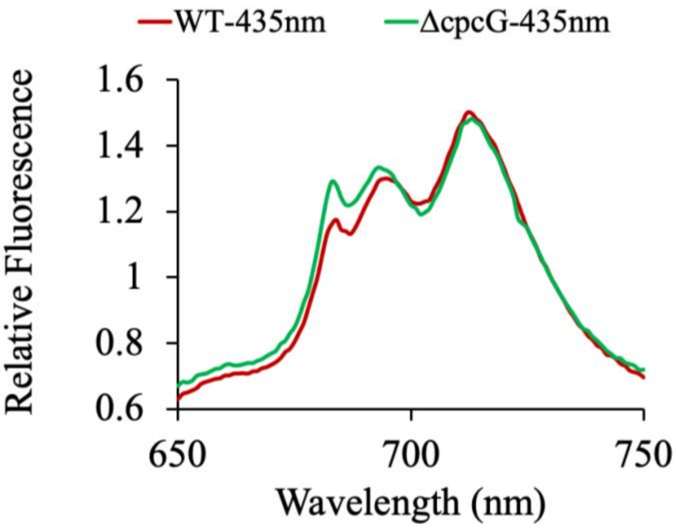

Fig. S5: 77K Fluorescence emission spectra of the WT, Δrrl and ΔcpcG strains when excited for phycocyanin at 580nm.

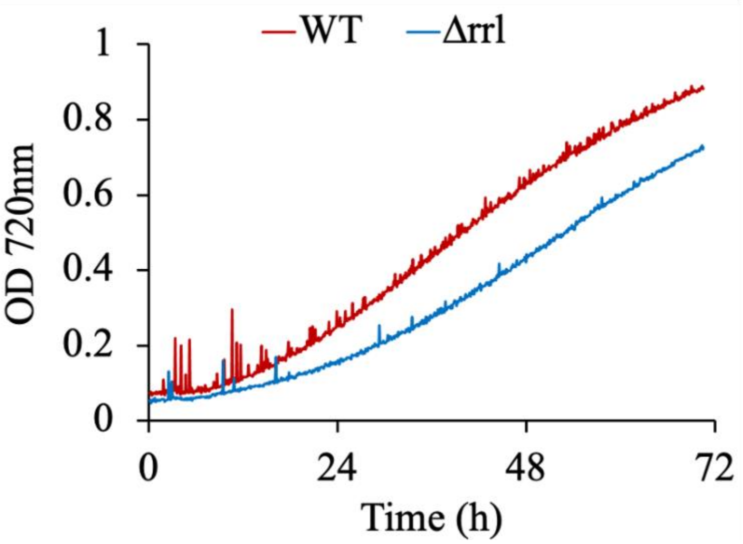

Fig. S6: Growth profiles of WT and Δrrl under 100 µmoles.m^{-2}.s^{-1} and HC

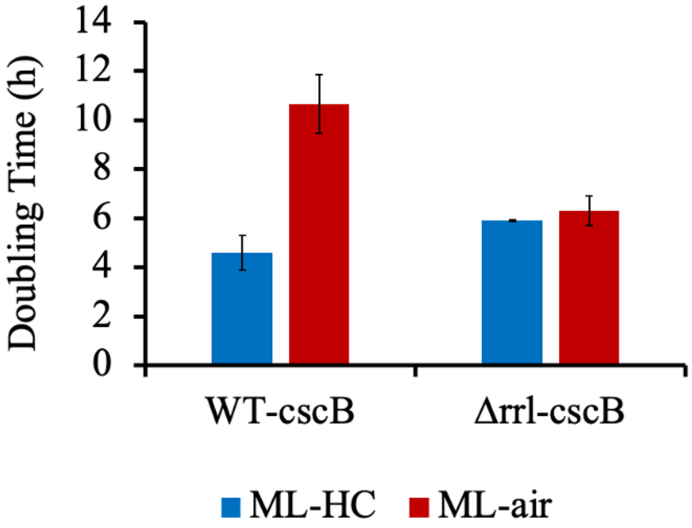

Fig. S7: Growth profiles of WT-cscB and Δrrl-cscB (sucrose producing strains)

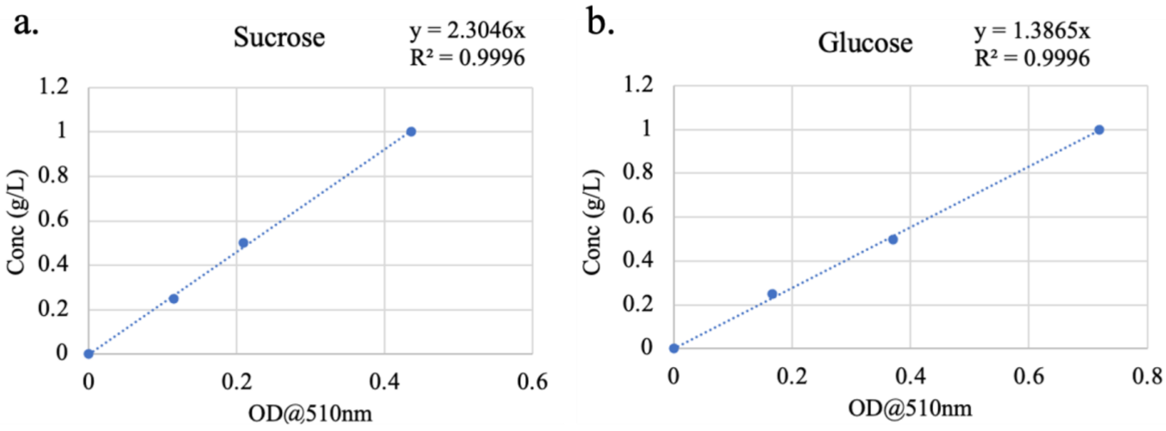

Fig. S8: Standard curves for (a) sucrose (b) Glucose estimated using the sucrose/D-glucose assay kit (Megazyme).

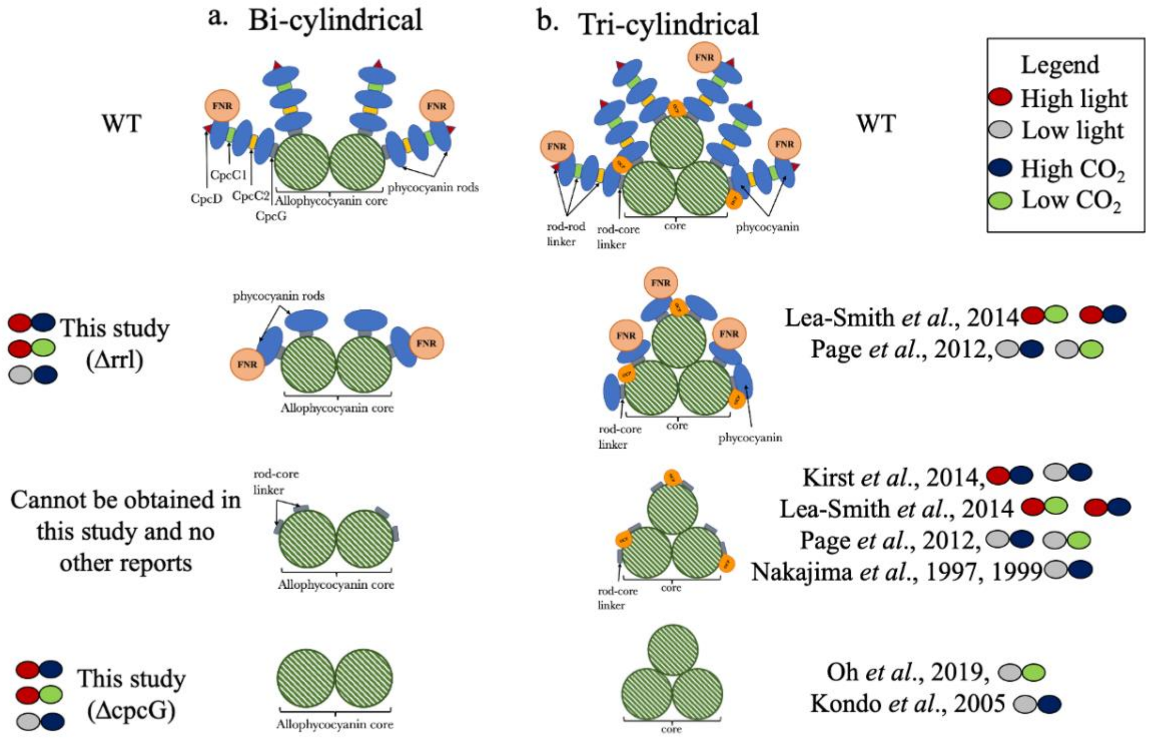

Fig. S9: A consolidated figure illustrating the structure of PBS with different degrees of antenna reduction for (a) bi-cylindrical and (b) tri-cylindrical core PBS. The references listed alongside the structure indicates the list of studies in the literature. The colored circles represents the growth conditions (light and CO_2) used in those reports. The legend for growth condition is provided in the figure, briefly, Red circle – high light (≥800 µmoles.m^{-2}.s^{-1}), grey circle – low light (≤100 µmoles.m^{-2}.s^{-1}), blue circle – high CO_2 (≥0.5 %) and green circle – low CO_2 (air).

Table S1: List of primers used in this study

NO.	PRIMER NAME	PRIMER SEQUENCE (5’ TO 3’)
**1**	gRNA-cpcG FW	agatgcgatttcatccgtgggctg
**2**	gRNA-cpcG-RV	agaccagcccacggatgaaatcgc
**3**	Upstream-cpcG FW	catttttttgtctagctttaatgcggtagttggtaccgcctgtcaattcagacgagtgaac
**4**	Upstream-cpcG-RV	atggttagggagtgacccgatcacgccaccggaattcaaccg
**5**	Downstream-cpcG FW	cggttgaattccggtggcgtgatcgggtcactccctaaccat
**6**	Downstream-cpcG-RV	gcccggattacagatcctctagagtcgacggtaccgccgctatctgcagcagttg
**7**	Locus-cpcG-FW	cgttacccccaaggcatccatg
**8**	Locus-cpcG-RV	gacatggtcatcctgccctacaag
**9**	RT-cpcG-fw	caccgttcgcgatttcatcc
**10**	RT-cpcG-rv	tcgctgttgagcagagtgtc
**11**	gRNA-cpcC-FW	GAAACggcactggtcaagcgctcggtgctcatgacgtaaG
**12**	gRNA-cpcC-RV	GCGACttacgtcatgagcaccgagcgcttgaccagtgccG
**13**	Tile1-FW	caggcaattggcgcagttcac
**14**	Tile1-RV	gcgttgatcccctagctgcca
**15**	Tile2-FW	cctgagagacctgggcttgaac
**16**	Tile2-RV	acaccttgcaacaggtcaaccg
**17**	Tile3-FW	cgctccactgtaatcgtgcgat
**18**	Tile3-RV	cagaactgatgctgcaaggccg
**19**	Confirmation-rrl-FW	gtgcctacagcaaaccatccatc
**20**	Confirmation-rrl-RV	gtgaactgcgccaattgcct
**21**	gRNA-cpcE-FW	GAAACccaatgcgcgtgcggcattgcgccgtaggggataG
**22**	gRNA-cpcE-RV	GCGACtatcccctacggcgcaatgccgcacgcgcattggG
**23**	gRNA-cpcB-FW	GAAACtgccaccgatgcaccgggcacgcccagagccaagG
**24**	gRNA-cpcB-RV	GCGACcttggctctgggcgtgcccggtgcatcggtggcaG
